# An empirical study on the intervention of traditional Chinese medicine five-element music in alleviating anxiety and depression among medical students: differences in effectiveness among three different musical instruments

**DOI:** 10.3389/fpsyg.2025.1625325

**Published:** 2025-09-29

**Authors:** Peng Lyu, Xiaobing Li, Xiyong Yao

**Affiliations:** ^1^College of Education, Chongqing College of International Business and Economics, Chongqing, China; ^2^The Youth League Committee, Chongqing Medical and Pharmaceutical College, Chongqing, China; ^3^College of Music, Mahasarakham University, Maha Sarakham, Thailand

**Keywords:** Traditional Chinese Medicine (TCM) five elements music, medical students, anxiety and depression, intervention efficacy, Guzheng

## Abstract

**Background:**

The efficacy of Traditional Chinese Medicine (TCM) Five Elements Music in intervening anxiety and depression has been extensively validated by trials. This study employed three different instruments to perform TCM Five Elements Music interventions on medical students’ anxiety and depression, aiming to evaluate the differences in therapeutic effects and provide empirical evidence for optimizing treatment protocols.

**Methods:**

A total of 148 medical students screened via the Self-Rating Anxiety Scale (SAS) and Self-Rating Depression Scale (SDS) were randomly divided into an Erhu group, Guzheng group, Bamboo flute group, and a Control group (37 participants each). The three intervention groups listened to TCM Five Elements Music for 15 min daily over 2 weeks, while the Control group received no intervention. Pre- and post-intervention assessments were conducted using the SAS and SDS.

**Results:**

Except for the Control group, all three intervention groups showed significantly lower SDS and SAS scores post-intervention compared to pre-intervention (*p* < 0.01). Statistically significant differences in SDS and SAS scores were observed among the three intervention groups post-intervention (*p* < 0.01), with the Guzheng group demonstrating the most pronounced intervention effect.

**Conclusion:**

TCM Five Elements Music effectively alleviates anxiety and depression in medical students. The therapeutic effects vary significantly across different instruments, with the Guzheng yielding the most remarkable outcomes.

**Clinical trial registration:**

This study has been registered with the International Traditional Medicine Clinical Trial Registry (Registration No.: ITMCTR2025000818).

## 1 Introduction

Anxiety and depression are common mental health challenges among college students, which in severe cases can even pose a threat to life (Mofatteh, 2020; Ramón-Arbués et al., 2020), causing irreparable losses to families and society. A survey of 9,779 college students across 6 Chinese universities revealed that the prevalence rates of depression and anxiety were 27.4 and 42% respectively, showing a continuous upward trend (Wang et al., 2022). To address this escalating mental health crisis, the Chinese government has prioritized reducing the prevalence of anxiety and depression in multiple national policy documents, including the *Healthy China Initiative (2019–2030)* issued by the State Council, the *Special Action Plan for Comprehensively Strengthening and Improving Mental Health Work Among Students in the New Era (2023–2025)* jointly released by 17 government departments (including the Ministry of Education), and the *Healthy China 2030 Blueprint* jointly issued by the CPC Central Committee and the State Council.

TCM Five Elements Music Therapy is a non-pharmacological intervention method proven effective in alleviating or improving anxiety and depression. Numerous studies have applied this therapy to diverse populations, such as pregnant and postpartum women (Liu et al., 2017; Wu et al., 2020), patients with severe conditions like stroke (Lin et al., 2017), diabetes (Dong et al., 2017), and cancer (Mastnak et al., 2020), as well as elderly individuals (He et al., 2021), yielding positive outcomes. Research has also confirmed the benefits of TCM Five Elements Music Therapy in mitigating anxiety and depression among medical students (Yao et al., 2024; Yao et al., 2025).

However, several questions regarding TCM Five Elements Music Therapy in the context of anxiety and depression intervention remain underexplored, such as: Does the choice of musical instrument influence the intervention efficacy? Which instrument demonstrates superior performance? To address these gaps, we designed a rigorous experiment to provide empirical evidence and develop a more precise intervention protocol for utilizing TCM Five Elements Music Therapy to alleviate anxiety and depression among medical students.

## 2 Participants and methods

### 2.1 Participants

#### 2.1.1 Inclusion criteria

Using the SAS and SDS, we screened sophomore medical students from a medical college in Chongqing for high levels of anxiety (SAS score ≥ 50) and depression (SDS score ≥ 53). After being fully informed about the study details, 148 students voluntarily participated.

#### 2.1.2 Exclusion criteria

Participants meeting any of the following conditions were excluded from statistical analysis due to invalid intervention:(1) Those who withdrew midway for any reason.(2) Those who did not follow the intervention requirements or failed to complete the post-intervention scale assessments.(3) Those who experienced major life events (e.g., bereavement) affecting emotional stability within the past month.

#### 2.1.3 Grouping method

Participants were randomly assigned to the Erhu group, Guzheng group, Bamboo flute group, or Control group using a random number table method, with 37 participants in each group. The four groups showed no statistically significant differences in grade, gender, age, anxiety/depression severity, or mean anxiety/depression scores (*p* > 0.05), indicating comparability across groups.

### 2.2 Intervention materials and methods

#### 2.2.1 Selection criteria for TCM five elements music tracks

China’s Traditional Wuxing (Five Elements) Therapeutic music (Zhengdiao), (Medium Tune) composed by renowned composer Shi Feng, recorded by the Central Conservatory of Music Chinese Music Ensemble, supervised by the Chinese Medical Association, and published by the China Medical Multimedia Press, is currently the most authoritative TCM Five Elements Music album (Zhou et al., 2023).

For this experiment, one piece from each of the five musical modes (Gong, Shang, Jue, Zhi, Yu) was selected from this album. The following audio processing was conducted using Logic Pro 10.8: Extraction of the main melody tracks from each piece. Trimming to 3-min core segments. Synthesis of the five mode segments into a 15-min coherent melody. Finally, three versions (Guzheng, Erhu, and Bamboo flute) were generated using a Traditional Chinese Musical Instrument Sound Library, with identical parameters (dynamics, tempo, etc.) maintained across versions except for timbre differences.

#### 2.2.2 Instruments used in the experiment

**Figure 1:**
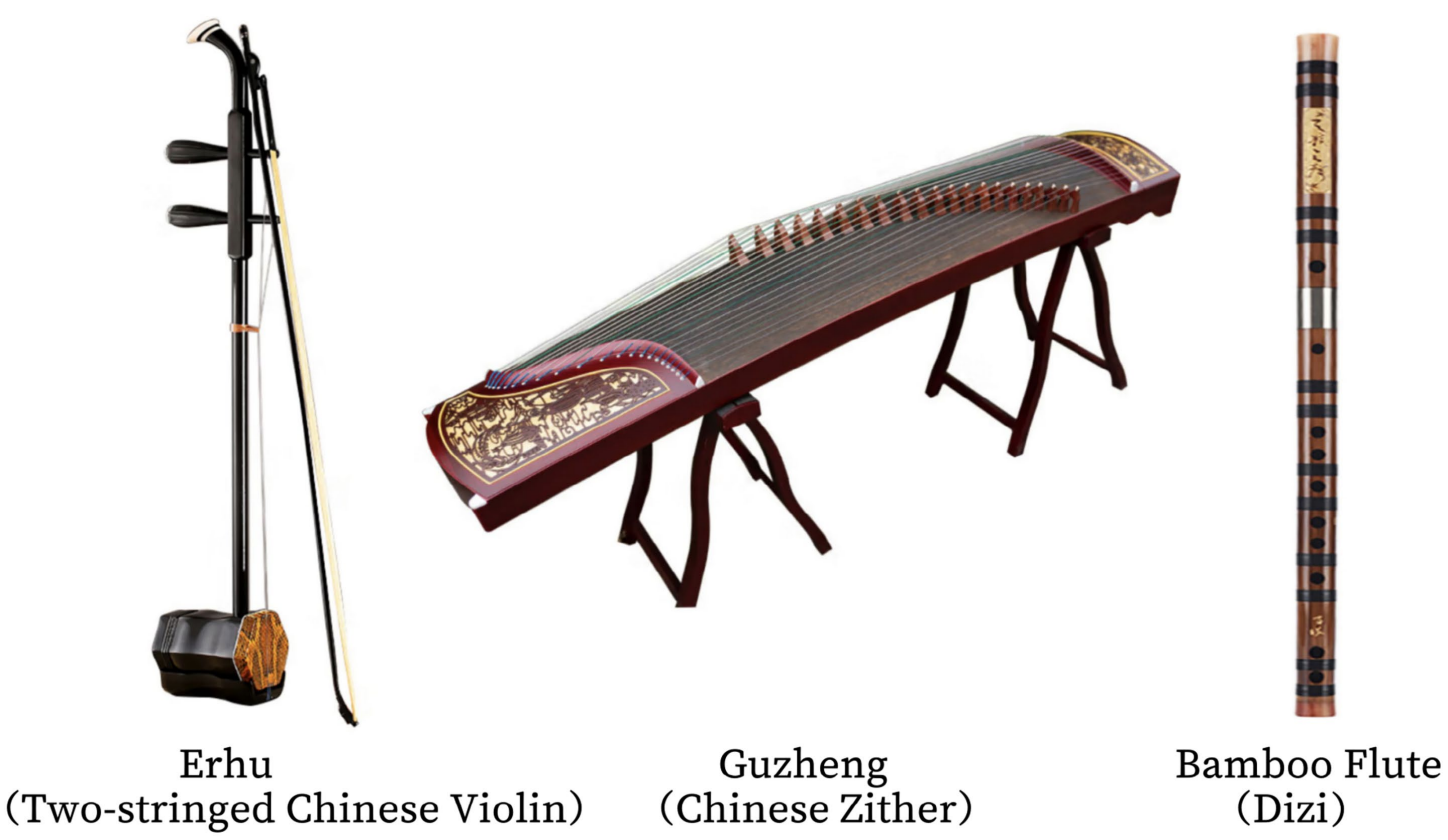
Three musical instruments used in the experiment.

Based on their sound production mechanisms, Traditional Chinese Musical Instruments are primarily categorized into four types: bowed string instruments, plucked string instruments, wind instruments, and percussion instruments. Percussion instruments, which are less suited for melodic performance, were excluded from this study. To comprehensively compare intervention effects across different instrument categories, representative instruments were selected from bowed string (Erhu), plucked string (Guzheng), and wind (Bamboo flute) categories, all of which are widely disseminated, highly accessible, and well-accepted in China. There are three musical instruments, as shown in Figure 1.

#### 2.2.3 Intervention protocol

The three intervention groups (Erhu, Guzheng, and Bamboo flute) listened to the experimental music for 15 min every night before bed over a two-week period. The Control group received no intervention. Social media groups were established for each cohort, with one supervising instructor assigned per group to guide and monitor adherence to the intervention protocols. Participants were required to log their daily completion status within the group.

### 2.3 Research instruments

The Self-Rating Depression Scale (SDS) (Zung, 1965) and the Self-Rating Anxiety Scale (SAS) (Zung, 1971), developed by Professor William W. K. Zung in 1965 and 1971 respectively, were employed as research instruments and outcome evaluation tools for this study. Both scales were administered before and after the intervention to assess therapeutic effects.

Questionnaire data collection was conducted through centralized self-administration. Participants completed the scales in class-based sessions, with supervising instructors providing standardized instructions to ensure accurate completion. After confirming participants’ understanding of the requirements, students independently completed the electronic questionnaires via smartphone by scanning the QR code for the Wenjuanxing platform. Upon completion, instructors verified questionnaire integrity and collected responses on-site.

### 2.4 Statistical methods

Data collection and organization were performed using Excel, while statistical analyses were conducted with SPSS 23.0 software. Normality tests indicated that SAS and SDS scores across all four groups before and after the intervention did not conform to a normal distribution (*p* < 0.05). Consequently, non-parametric tests (Kruskal-Wallis H tests for multiple independent samples) were employed for intergroup comparisons.

## 3 Results

A total of 148 questionnaires were distributed prior to the intervention, with 145 valid questionnaires retrieved post-intervention (3 participants dropped out).

### 3.1 Overall pre- and post-intervention outcomes

Kruskal-Wallis H tests (non-parametric tests for multiple independent samples) were conducted to compare pre- and post-intervention SAS/SDS scores. Results revealed significant intergroup differences: for SAS scores, *χ*^2^ = 25.221, *p* < 0.001; for SDS scores, *χ*^2^ = 27.054, *p* < 0.001. Detailed statistics are presented in Table 1.

**Table 1:** Check the statistical results table a, b.

Statistical methods	Post-intervention SAS scores	Post-intervention SDS scores	Pre-intervention SAS scores	Pre-intervention SDS scores
Chi-square	25.221	27.054	1.746	3.094
Degree of freedom	3	3	3	3
Asymptotic significance	0.000	0.000	0.627	0.377

a. Kruskal Wallis test; b. variable grouping (grouping).

### 3.2 Comparison of SAS scores among four groups: pre- and post-intervention

Prior to the intervention, the median SAS scores of all four groups exceeded 50 points, with no statistically significant differences observed among groups (*F* = 0.299, *p* = 0.826). Post-intervention, the median SAS scores of all four groups decreased by more than 2 points, with intervention groups dropping to the 40s range. The intergroup differences became statistically significant (*F* = 8.654, *p* < 0.0001). Notably, the Guzheng group demonstrated the largest reduction, with a median score decline of 8.750 points (*t* = 10.290, *p* < 0.0001). Detailed statistics are provided in Table 2.

**Table 2:** Comparison of differences in students’ SAS scale scores pre- and post-intervention across different intervention groups.

Group	*N*	Pre-intervention SAS scores M(Q25, Q75)	Post-intervention SAS scores M(Q25, Q75)	Comparison of M Pre- and Post-Intervention	*t*	*P*
Guzheng Group	37	53.750(51.250, 56.250)	45.000(41.250, 48.750)	−8.750	10.290	0.000
Erhu Group	37	53.750(51.250, 55.000)	46.250(41.875, 55.000)	−7.500	4.894	0.000
Bamboo flute group	37	52.50(51.25, 57.50)	48.750(46.250, 52.625)	−3.750	5.639	0.000
Control group	37	55.00(50.00, 57.50)	52.500(48.750, 56.875)	−2.500	1.290	0.205
*F*		0.299	8.654			
*P*		0.826	0.000			

SAS scores were non-normally distributed and are presented as M (Q), i.e., median (interquartile range).

**Figure 2:**
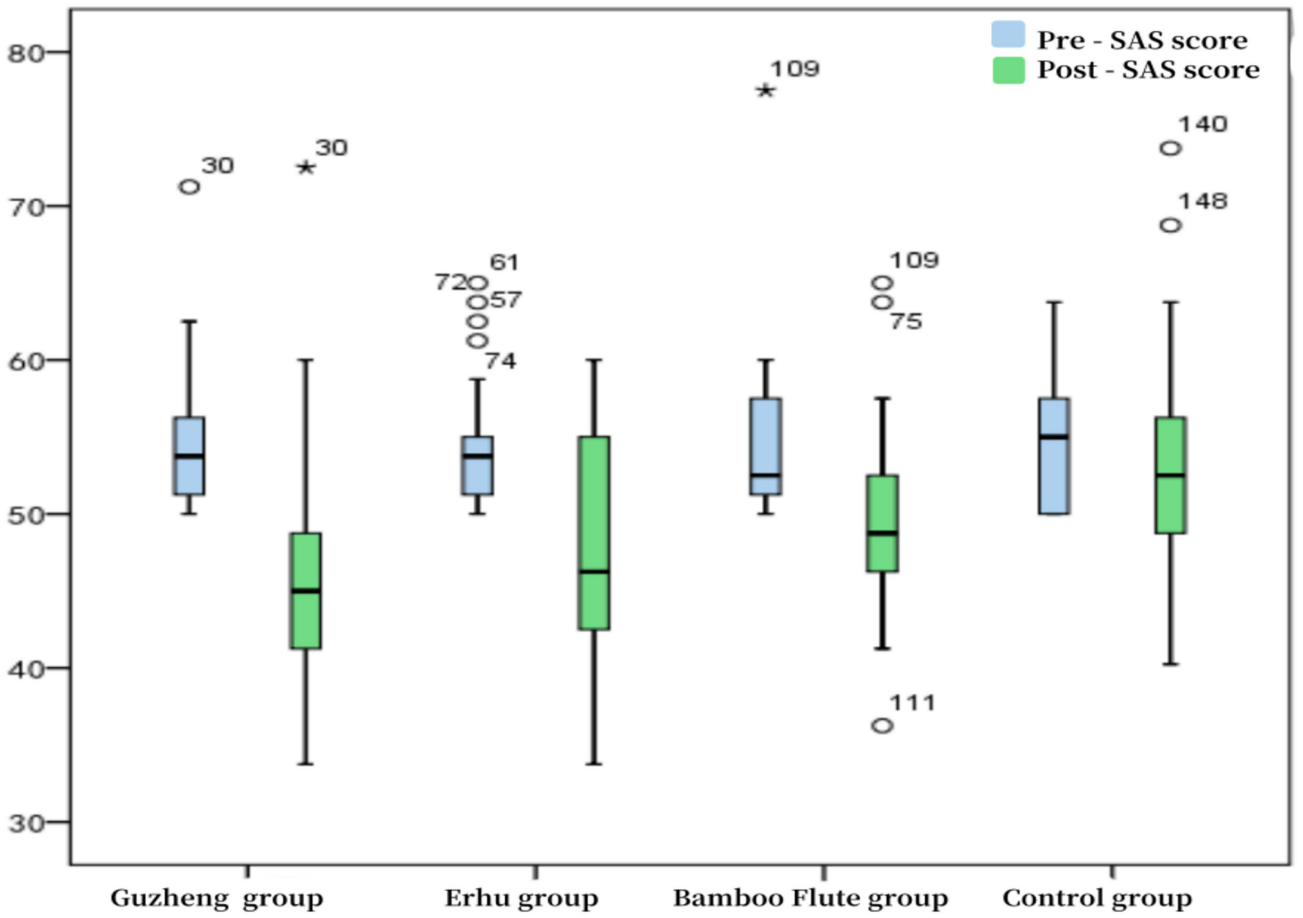
Box plot of students’ SAS scale scores pre- and post-intervention.

Analysis of intervention efficacy differences across instrument groups revealed that the Guzheng group demonstrated the most significant reduction in SAS scores, with a median decrease of 8.750 points (*p* < 0.0001) post-intervention compared to pre-intervention. This was followed by the Erhu and Bamboo flute groups (see Figure 2).

### 3.3 Comparison of SDS scores among four groups: pre- and post-intervention

Prior to the intervention, the median SDS scores of all four groups exceeded 55 points, with no statistically significant differences observed among groups (*F* = 1.683, *p* = 0.173). Post-intervention, except for the Control group, the median SDS scores of the remaining three intervention groups decreased by more than 6 points, with statistically significant intergroup differences (*F* = 9.318, *p* < 0.0001). Detailed statistics are provided in Table 3.

**Table 3:** Comparison of differences in students’ SDS scale scores pre- and post-intervention across different intervention groups.

Group	*N*	Pre-intervention SDS scores M(Q25, Q75)	Post-intervention SDS scores M(Q25, Q75)	Comparison of M Pre- and Post-Intervention	*t*	*P*
Guzheng Group	37	56.250 (53.750, 60.000)	46.250 (43.125, 49.375)	−10.000	6.531	0.000
Erhu Group	37	57.500 (55.000, 60.000)	47.500 (43.750, 53.125)	−10.000	5.476	0.000
Bamboo flute group	37	56.250 (51.250, 59.375)	50.000 (46.250, 57.500)	−6.250	2.857	0.007
Control group	37	57.500 (54.375, 61.250)	58.750 (50.625, 60.000)	1.250	1.671	0.103
*F*		1.683	9.318			
*P*		0.173	0.000			

SDS scores were non-normally distributed and are presented as M (Q), i.e., median (interquartile range).

**Figure 3:**
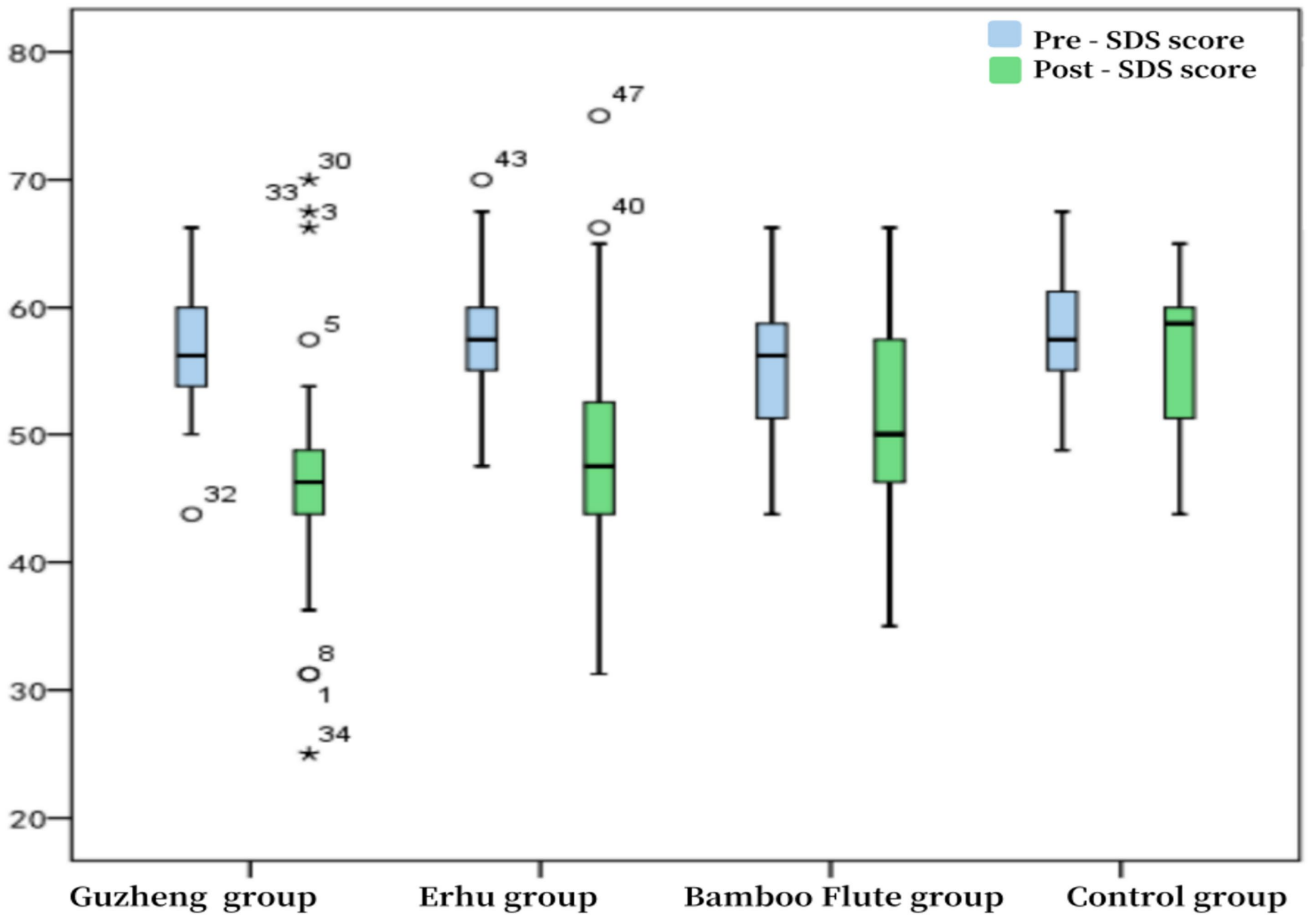
Box plot of students’ SDS scale scores pre- and post-intervention.

Analysis of intervention efficacy differences across instrument groups revealed that the Guzheng and Erhu groups demonstrated the most significant reductions in SDS scores, with median scores decreasing by 10 points (*p* < 0.0001). The reduction in scores was statistically significant, followed by the Bamboo flute group (see Figure 3).

## 4 Discussion

### 4.1 Significant effects of TCM five elements music therapy on alleviating anxiety and depression

The Huangdi Neijing (The Yellow Emperor’s Inner Canon), written over two millennia ago, documented the therapeutic application of TCM Five Elements music theory in disease treatment—a traditional Chinese method for emotional regulation (Meng and Wang, 2017). Similarly, the Records of the Grand Historian: Book of Music states, “Music can invigorate the bloodstream, harmonize the spirit, and rectify the mind” (Zhang and Zhang, 2014), suggesting its therapeutic potential for mental disorders. Modern medical theories parallel this concept (Davis et al., 2008), proposing that appropriate music induces synchronous resonance in human tissues, thereby regulating physiological rhythms such as pulse, respiration, and heart rate to achieve a state of harmony (Rolvsjord and Stige, 2015). Furthermore, music’s acoustic energy exerts benign stimulatory effects on the central nervous and endocrine systems (Xiong et al., 2010). Numerous studies have demonstrated that TCM Five Elements music therapy offers notable advantages in addressing anxiety and depression among university students, including efficacy, safety, cost-effectiveness, ease of implementation, and high patient acceptability—meriting broader clinical adoption (Bradt et al., 2022; Chen, 2018; Chen et al., 2015).

In this study, post-intervention SAS and SDS scores of participants were significantly lower than pre-intervention scores (both *p* < 0.0001), indicating marked improvements in anxiety and depression symptoms. These findings align with prior research by Zhang et al. (2018) and Cao et al. (2024).

### 4.2 Differential intervention effects of musical instruments on anxiety and depression

Post-intervention comparisons revealed that the Guzheng group exhibited the most pronounced reductions in both SAS and SDS scores (both *p* < 0.0001), followed by the Erhu group, while the Bamboo flute group showed lesser though still significant improvements. These outcomes suggest that variations in instrumental timbre and acoustic properties may contribute to differential therapeutic effects on emotional regulation—a hypothesis warranting further investigation.

This study compared pre- and post-intervention SAS scores to evaluate the anxiety-alleviating effects of different instruments. Results showed significant reductions in SAS scores across all three intervention groups (Guzheng, Erhu, and Bamboo flute) (*p* < 0.001). Post-intervention, the SAS scores of all three intervention groups were significantly lower than those of the Control group (*p* < 0.001), with the Guzheng group demonstrating the most pronounced reduction (*p* < 0.001), followed by the Erhu and Bamboo flute groups. These findings indicate that TCM Five Elements music interventions effectively reduced anxiety among medical students, with notable variations in efficacy across instruments (Guzheng > Erhu > Bamboo flute).

Similarly, SDS score comparisons revealed significant reductions in depressive symptoms across all three intervention groups (*p* < 0.001). Post-intervention SDS scores were significantly lower in the intervention groups compared to the Control group (*p* < 0.001), with the Guzheng group outperforming both the Erhu and Bamboo flute groups (*p* < 0.05). This suggests that while TCM Five Elements music interventions improved depressive symptoms, the choice of instrument significantly influenced outcomes, with the Guzheng demonstrating superior efficacy.

## 5 Conclusion

In summary, this study reaffirms our team’s previous findings that TCM Five Elements music effectively alleviates anxiety and depression in medical students (Yao et al., 2024). Furthermore, it highlights significant variations in intervention efficacy across different instruments, with the Guzheng demonstrating more pronounced effects compared to the Erhu and Bamboo flute. These results provide a more precise approach to applying TCM Five Elements music for mitigating anxiety and depression in this population.

However, this study has several limitations. First, while the selected instruments (Guzheng, Erhu, and Bamboo flute) are widely recognized and culturally accepted in China, individual preferences for specific instruments were not considered, which may have influenced outcomes. Second, although three representative instruments were included, other instruments might yield superior results and warrant investigation. Third, uncontrolled variables such as differences in listening volume or environmental conditions during music exposure could have affected results. Future research should address these limitations to further optimize intervention protocols.

## Data Availability

The original contributions presented in the study are included in the article/supplementary material, further inquiries can be directed to the corresponding author/s.
